# Supplementary material for: Controlling a robotic arm for functional tasks using a wireless head-joystick: A case study of a child with congenital absence of upper and lower limbs
Source: PLoS One. 2020 Aug 5;15(8):e0226052. doi: 10.1371/journal.pone.0226052 (PMC7406178; doi:10.1371/journal.pone.0226052)
Supplement: Attachment — Submitted filename: PLos ONE Review Response.docx [file pone.0226052.s004.docx]

**Response to Editors and Reviewers**

We thank the Editor and all 3 reviewers for their insightful comments. We have addressed these concerns with significant changes in the manuscript as seen below. We think these changes have greatly improved the manuscript and hope that the revised version is suitable for publication.

We have provided a point-by-point rebuttal to each comment below. For the sake of clarity, we have color coded the text as follows:

Editors and Reviewer comments in BLACK

Authors’ response in BLUE

Corresponding changes in manuscript in GREY

-------

**Journal Requirements:**

1. When submitting your revision, we need you to address these additional requirements.

Please ensure that your manuscript meets PLOS ONE's style requirements, including those for file naming. The PLOS ONE style templates can be found at

http://www.journals.plos.org/plosone/s/file?id=wjVg/PLOSOne_formatting_sample_main_body.pdf and http://www.journals.plos.org/plosone/s/file?id=ba62/PLOSOne_formatting_sample_title_authors_affiliations.pdf

We have made edits throughout our manuscript to ensure that our manuscript meets PLOS ONE’s style requirements

2. Please include captions for your Supporting Information files at the end of your manuscript, and update any in-text citations to match accordingly. Please see our Supporting Information guidelines for more information: http://journals.plos.org/plosone/s/supporting-information.

We have included captions for supporting information files at the end of the manuscript,

Supporting information

Video S1. Example drinking task for main participant. In this trial, the main participant conducts a complete drinking task. The robotic arm is initialized to the starting position after which the participant commands it to move towards the cup, grasp the cup, and then move and orient the cup such that he is able to drink from the straw.

Video S2. Example stacking task for main participant. In this trial, the main participant conducts a complete stacking task. The robotic arm is initialized to the starting position after which the participant commands it to approach, grasp, orient, and place each block on the preceding block or on the table in the case of the first block.

Data File S3. Data from participant trials as used for analysis. Included are the movement times and dimensionless jerk values for each of the main and control participants’ drinking and stacking trials.

3. We note that Figure 1 and your videos includes an image of a [patient / participant / in the study].

As per the PLOS ONE policy (http://journals.plos.org/plosone/s/submission-guidelines#loc-human-subjects-research) on papers that include identifying, or potentially identifying, information, the individual(s) or parent(s)/guardian(s) must be informed of the terms of the PLOS open-access (CC-BY) license and provide specific permission for publication of these details under the terms of this license. Please download the Consent Form for Publication in a PLOS Journal (http://journals.plos.org/plosone/s/file?id=8ce6/plos-consent-form-english.pdf). The signed consent form should not be submitted with the manuscript, but should be securely filed in the individual's case notes. Please amend the methods section and ethics statement of the manuscript to explicitly state that the patient/participant has provided consent for publication: “The individual in this manuscript has given written informed consent (as outlined in PLOS consent form) to publish these case details”.

If you are unable to obtain consent from the subject of the photograph, you will need to remove the figure and any other textual identifying information or case descriptions for this individual.

We have acquired from the participant’s guardian a signed Consent Form for Publication in a PLOS Journal and it has been mentioned in the manuscript.

The individual in this manuscript has given written informed consent (as outlined in PLOS consent form) to publish these case details.

4. We note you have included a table to which you do not refer in the text of your manuscript. Please ensure that you refer to Table 1 in your text; if accepted, production will need this reference to link the reader to the Table.

We have referred to all tables present in our manuscript within the text.

**Reviewer's Responses to Questions**

5. Review Comments to the Author

Please use the space provided to explain your answers to the questions above. You may also include additional comments for the author, including concerns about dual publication, research ethics, or publication ethics. (Please upload your review as an attachment if it exceeds 20,000 characters)

Reviewer #1: The paper presents an evaluation of a "head joystick" (IMU-based head motion tracker) used to control a robotic arm by a child with a congenital absence of all four limbs as well as a control participant. They demonstrate that the participant was able to use the head joystick to effectively control the arm and accomplish two different tasks. Overall, I believe that the paper is well-written and represents an appropriate first evaluation of the system. I have no major concerns with it, and my suggestions mostly have to do with improving clarity and informativeness.

We thank the reviewer for the positive comments

1. Please clarify whether the participant's prior two studies with your group also involved the head joystick.

The prior studies involved control with the shoulder and torso movements, not the head joystick. This has now been clarified in the manuscript.

These prior studies involved the control of these devices using shoulder and torso movements.

2. Please describe the "some unstructured sessions" in more detail. How many sessions? Approximate time per session? Approximate activities performed?

The following text has been included to describe the unstructured sessions:

Initially, there were 4 unstructured sessions, each lasting about 30-45 minutes in length. We used these sessions to calibrate the interface to make sure that the head movements performed were in a comfortable range when controlling the robot. During each session, the participant was asked to do some exploratory movements of the head to understand how movements of each DOF controlled the robot, and to learn the operation of the switches (which was used to toggle between the translation/orientation modes). In addition, the participant was also free to perform any tasks of their liking using the robot arm like trying to pick up an object from a table.

3. Please consistently put spaces between the number and unit (e.g., 0.05 m/s, not 0.05m/s).

Thank you. This has been corrected.

4. While photos of the two tasks are provided, I would have appreciated some more information about the parameters of the task - exact distances to be covered, precision required etc.

We thank the reviewer for this suggestion. We have now included these details in the methods section. Table 1 has the dimensions of the stacking blocks.

The robotic arm was always initialized in the same starting position (X: 0.40 m, Y: 0.30 m, Z: 0.30 m from the base of the robot with the gripper open enough to grasp the cup and facing towards the right) while the cup was in front of and to the right of the participant on the table (mean X: 0.58 m, mean Y: 0.25 m, Z: 0.00 m) - see Fig 1c. [...] The final position of the grasper holding the cup when the main participant took a drink was (mean X: 0.35 m, mean Y: -0.04 m, Z: 0.10 m) with the grasper facing towards the participant.

The location of the tower was chosen by the participant (mean X: 0.39 m, mean Y: 0.28 m) to allow them to have a clear vision of the remaining blocks for the remainder of the tasks.

| Block | Outer dimension of closed face (mm) | Inner dimension of open face (mm) |
| --- | --- | --- |
| 1 | 44 | 54 |
| 2 | 46 | 61 |
| 3 | 53 | 68 |
| 4 | 60 | 77 |
| 5 | 67 | 84 |

Table 1. Dimensions of stacking blocks. The difference between the inner dimension of the upper block and the outer dimension of the lower block defined the precision to which the block had to be placed to be secure.

The blocks were placed directly across the table from the participant in five orientations not matching the target orientation - see Fig 1d. The first block had its opening facing upwards; the second block had its opening facing away from the participant; the third block had its opening facing towards from the participant; the fourth block had its opening facing to the right of the participant; the fifth block had its opening facing to the left of the participant. These starting positions were standardized throughout trials (position of first block with respect to the base of the robot: mean X: 0.31 m, mean Y: 0.49 m).

5. I feel like more results could have been provided. In the current form, the results are limited to task completion times and percentage of successfully completed tasks. Would have been useful to see more information about any false positives/negatives during switching, task completion strategies, motion smoothness, etc. Perhaps even subjective information like user satisfaction.

We thank the reviewer for this suggestion. We have now included figures for the jerk calculations (quantifying smoothness) for both drinking and stacking tasks. The results are similar to that seen in the movement times (smoothness increases overall with learning as evidenced by decreased jerk).

Because the control was continuous, the only ‘discrete’ errors we could see were during grasping (which were controlled by switches). The number of grasping errors was quite low and we did not observe any trends with practice even as movement times decreased. We also did not observe any major qualitative changes in task completion strategies.

We do not have any standard measures of perceived usability or satisfaction. However, we the fact that the participant continued this task for over 3 months and was enthusiastic about returning for future visits is a potential indicator that he was satisfied with the interface. We have added this in the Discussion

Moreover, although we had no direct measures of user satisfaction, the fact that the participant continued this task for over 3 months and was enthusiastic about returning for future visits is a potential indicator that he was satisfied with the interface.

These changes related to the dimensionless jerk have been incorporated in the Data analysis and Results of the manuscript as follows:

In the Data analysis:

## **Dimensionless jerk.** To quantify the smoothness of the movement, we computed the dimensionless jerk values for the tasks from the end-effector position data on each trial. The jerk was normalized by the time and peak velocity to yield a dimensionless measure which has been shown to be more appropriate measure The position values were first low pass filtered using a 2^nd^ order Butterworth filter with a cutoff frequency of 6 Hz. The jerk values for each trial were then calculated from subsequent derivatives of the filtered position data and integrated over the duration of the trial. The dimensionless jerk value was then computed as follows

$$Dimensionless Jerk= \sqrt{\left( \int_{t_{1}}^{t_{2}} \left\| \overset{⃛}{x} \right\|^{2}dt \right){MT}^{3}/v_{peak}^{2}}$$

where $\overset{⃛}{x}$ indicates the instantaneous jerk, $MT=\left( t_{2}- t_{1} \right)$ is the movement time of the trial, and $v_{peak}$ indicates the magnitude of the peak velocity during the trial.

In the Results:

## **Dimensionless jerk**

### **Drinking task**

The dimensionless jerk of the main participant closely followed the pattern of the movement times – see Fig 7. Two trials on visit #5 (trials marked with an “x” on Fig 5) involved a lot of talking during the trial and a poorly aligned IMU. Additionally, the data for trial #15 was corrupted and therefore omitted from the jerk analysis. Comparing the dimensionless jerk values from the first four trials to the last four trials shows a 45% decrease in dimensionless jerk value.

### **Stacking task**

The dimensionless jerk of the main participant for the stacking trials also closely followed the pattern of the movement times – see Fig 8. Comparing the jerk values from the first three completed trials (trials 2, 5, and 8 were incomplete) to the last three completed trials shows an 57% decrease in dimensionless jerk value.

Figure 7. Dimensionless jerk values for drinking task trials for the main participant across practice. Jerk times denoted with a “x” were those in which the main participant had significant distractions or poor IMU alignment which led to large jerk values. Additionally, trial #15 is omitted due to data corruption.

Figure 8. Dimensionless jerk values for stacking task trials for the main participant across practice. Trials 3, 5, and 8 were incomplete in which the main participant was unable to successfully place the last block due to dropping it outside the reach of robotic arm.

Reviewer #2: The authors present a study conducted with a child with congenital limbs absence controlling a 7dof robotic arm (Jaco, Kinova) using a head-based IMU system. The goal was to demonstrate that the IMU-based head joystick is well suited for the control of the robotic arm and that the system allowed the child to reach a level of handiness comparable to the one of an unimpaired individual controlling the same robotic arm with a manual joystick.

The manuscript is well organized and well written so that also non-specialists can understand the work. The state of the art is clear and sound. Details of the methodology are sufficient to allow the experiments to be reproduced and the original data are accessible.

We thank the reviewer for the positive comments

My main concern is about the comparison between the main subject (child with congenital limb absence) and the control subject. If the goal is to examine the use of an IMU-based controlling system for a robotic arm, I think it would be more fare to compare the performance of the same subject using two different control systems. So in this case I would have preferred to see the main subject practicing with the IMU-based and with the commercially available head-controlled joystick. Or have the control subject practicing and performing the tasks with the joystick of the Jaco and with the IMU-based joystick. My suggestion is to add a group of healthy subjects, not only one, that are controlling the robot with both modalities. And then also present the case study with the congenital lack of limbs.

We thank the reviewer for the comment. We want to emphasize that the purpose of including the control participant was not to compare the IMU interface directly with a head joystick (there were no statistical comparisons being made), but to only provide a baseline reference for the movement times (otherwise the magnitude of the movement times would not be directly interpretable).

An important novel contribution of the current study was to examine the feasibility of the interface in children with movement impairment and show that they can accomplish complex tasks in a reasonable period of time. Therefore, we think that adding a group of healthy adult participants doing both interfaces (which could provide information about the relative usability of the interfaces) is not directly related to the current focus of the manuscript. The reviewer’s point about directly comparing with a commercially available head joystick is well taken and while this is not currently feasible, this line of investigation certainly lies within the scope of our future work.

We have now added this line to clarify the use of the control participant.

It is important to note that the control participant was an adult controlling a manual joystick; therefore, these data are not intended to be a direct comparison with the child using the head-joystick. Rather, given that the tasks we used were complex tasks for which benchmarks are not already available, the data from the control participant provide reference values that help in interpretation of the magnitudes of the change in performance with learning and the final performance level achieved by the child.

Minor concerns:

- It would be nice to have an idea (on average) of how long the free exploration during each

session was.

The following text has been included:

The free exploration lasted anywhere between 3-5 minutes. The goal of the free exploration was just to ensure that the interface was working as intended and the participant was ready to start controlling the robot arm.

- There is no reference in the manuscript to Table 1.

We thank the reviewer for pointing this out. This is now Table 2 and is referenced in the manuscript.

The amount and distribution of practice for both participants is shown in Table 2.

- When the authors report the average movement time, I suggest reporting also the standard

Deviation

We have included standard deviation next to their corresponding means in the text. We have also included the individual data points in the figures

Reviewer #3: The paper described a case study where a child with congenital absence of all four limbs controlled a robotic arm using custom head movement control. However, the paper had a number of issues.

• The authors did not provide a comprehensive review of the existing work on control interfaces for assistive robotic manipulators. Only brain control was mentioned. JACO arms could be controlled with wheelchair joystick, head control, sip-and-puff, and head array system. In addition, there are many types of custom control interfaces (e.g., voice control and eye gaze control) in existing literature. It was unclear how the proposed approach is more advantageous than existing work.

• The novelty or focus of the study is not clearly stated. From the technical perspective, as mentioned earlier, JACO arm could be controlled with wheelchair joystick, head control, sip-and-puff, and head array system, so it was not clear how the head control system used in this study differed from the JACO’s existing capability.

We thank the reviewer for raising this point. In the introduction, we compare our method in the context of existing ‘head control’ methods (head arrays and head joysticks) as the focus is on developing interfaces with individuals with severe impairments (where the assumption is that that they cannot use a regular manual joystick). Here, one main advantage of our method is to create a wireless ‘continuous’ control interface like the joystick for high DOF tasks (which is more intuitive compared to head arrays which rely on switches).

In the discussion, we have now talked about other interfaces (such as the sip and puff system, eye gaze control, voice commands). One primary advantage of our interface is that it is designed to be flexible and used for high-DOF control with precision. The relative advantages of each system will depend to a great extent on the abilities of the individual (e.g. a more severely impaired individual with limited head movements may benefit from a voice controlled or sip-and-puff system). We have included this text in the Discussion

In addition to head control methods discussed here, several alternate control interfaces to manual joysticks have been developed for individuals with severe movement impairments. These include sip-and-puff systems, voice control [23], gaze control [24] and tongue control [25]. These interfaces typically involve some tradeoff between (i) the number of control dimensions (e.g., a device that only allows control of 1 or 2 dimensions would require frequent ‘switching’ to control a high DOF robotic arm), (ii) the type of control (e.g., discrete controls are possible using voice commands but are less intuitive and precise relative to continuous control like a joystick), and (iii) the ‘invasiveness’ of the device both in terms of its physical attributes (e.g., whether it is easily wearable, wireless etc.) but also how it affects other activities such as communication (e.g., gaze or voice based controls may interfere with natural day-to-day behavior). Ultimately, the choice of the interface will depend both on existing movement abilities for the individual and the number of degrees of freedom to be controlled.

From the clinical perspective, only one subject participated in the study and the training/evaluation protocol was somewhat unstructured, and thus cannot be generalized.

This is a highly unique population (child with no movement of the limbs)- so the goal was to provide proof of concept that the head IMU interfaces are feasible for complex movements in children. We have added this as a limitation of the study.

Although conducted as a case study, which places limits on generalizability, our results add to these prior findings by demonstrating that (i) these interfaces are well-suited for children, and (ii) the improvement in performance over multiple practice sessions is substantial (up to 40-50% reduction in movement times) and comparable to manual joystick performance.

In addition, only task completion time was reported, and user perceived usability was not mentioned. Given the nature of such intervention, it would be helpful to know the user feedback on the control interface and training procedure.

The reviewer makes a good point but we do not have any standard measures of perceived usability or satisfaction. However, the fact that the participant continued this task enthusiastically for over 3 months is an indicator that he was satisfied with the interface.

• The performance contrast between the case subject and control subject is not well justified, as the performance could be affected by not only the input devices (head vs hand), but also their personal characteristics including but not limited to physical limitations. Thus, it is unclear how such information would be clinically or practically meaningful.

As in our response to reviewer 2, the performance of the control subject was only to provide a baseline reference for the movement times (and not to directly compare them). We have now added this line to clarify the use of the control participant.

Given that the control participant was an adult controlling a manual joystick, these values are not intended to be a direct comparison with the child using the head-joystick. Rather, given that the tasks we used were complex tasks for which benchmarks are not already available, the data from the control participant provide reference values that help interpretation of the magnitudes of the change in performance over learning and the final performance level achieved by the child.

• The limitation of the control interface was not stated. How does head movement control affect visual feedback a user would need for accurately controlling the arm motion? How generalizable the approach is when used in real-life situations (e.g., wheelchair-mounted arm)?

Both tasks required visual feedback since they required precision to hold the cup or stack the blocks. The head movements performed were quite small; because head movements directly controlled the velocity of the robot, big movements of the head were not needed. Additionally, when big movements of the robot were needed (where visual feedback is not as critical). So at least to a first approximation, these movements did not seem to affect the use of visual feedback to control the movement of the robot arm. Even when it is mounted on a wheelchair, it is unlikely that the user will do both wheelchair navigation and robot arm control simultaneously; therefore, we do not think that the head movements will pose a problem in real-world situations.

We have referred to this in the Discussion

It is also worth noting that despite requiring the use of head movements to control the robot to precisely control the end effector, the child was able to successfully perform these tasks, indicating that the small head movements performed did not interfere greatly with the use of visual feedback..

We refer to the limitations of the current approach in the penultimate paragraph in the Discussion. (Burden of learning being completely on the user, and the exclusive use of head signals)

In terms of further improvements to our design, we wish to highlight two issues. First, a limitation of our approach is that the ‘burden of learning’ is all on the user. This may be especially challenging for children, who show deficits relative to adults in learning such interfaces [[21], [26]](https://www.zotero.org/google-docs/?b6GAIs). One way to improve this is to use either an adaptive interface that adjusts to the user [[27], [28]](https://www.zotero.org/google-docs/?Hvl6lc), or use a shared control framework so that the autonomy of control can be shared between the human and the machine [[29]](https://www.zotero.org/google-docs/?8ut36B). Second, for the sake of simplicity, we relied only on head movements (i.e. kinematics) to control the device. However, in the control of other neuroprosthetics, electromyographic signals from different muscles is often used to augment the movement repertoire by providing distinct control signals for the control of the external device [[30]–[32]](https://www.zotero.org/google-docs/?GplkVP). Therefore, a hybrid combination of IMU signals along with electromyography may further facilitate the user for efficient control of high DOFs [[33]](https://www.zotero.org/google-docs/?2NHKTt).

We have also added this line to highlight the goal of responding in real-life situations

Addressing these limitations could increase the potential of this approach to deal with real-life situations which require both speed and accuracy.
