# Supplementary material for: Controlling a robotic arm for functional tasks using a wireless head-joystick: A case study of a child with congenital absence of upper and lower limbs
Source: PLoS One. 2020 Aug 5;15(8):e0226052. doi: 10.1371/journal.pone.0226052 (PMC7406178; doi:10.1371/journal.pone.0226052)
Supplement: Attachment — Submitted filename: PLos ONE Response to Reviewers 2.docx [file pone.0226052.s005.docx]

**Response to Editors and Reviewers**

We thank the Editor and the reviewers for their insightful comments. We have addressed these concerns with changes in the manuscript as seen below. We think these changes have improved the manuscript and hope that the revised version is suitable for publication.

We have provided a point-by-point rebuttal to each comment below. For the sake of clarity, we have color coded the text as follows:

Editors and Reviewer comments in BLACK

Authors’ response in BLUE

Corresponding changes in manuscript in GREY

-------

**Journal Requirements:**

N/A

**Reviewer's Responses to Questions**

6. Review Comments to the Author

Please use the space provided to explain your answers to the questions above. You may also include additional comments for the author, including concerns about dual publication, research ethics, or publication ethics. (Please upload your review as an attachment if it exceeds 20,000 characters)

Reviewer #1: (No Response)

Reviewer #2: I want to tank the authors for the detailed replies to all my concerns. Everything is good with the exception of the main concern. I am still not fully convinced about the lack of a control group. I understand that comparing the IMU-based interface with the commercial available interface is out of the scope of this specific paper, and I am okay with that. As the author said themselves “the purpose of including the control participant was to only provide a baseline reference for the movement times” but with n=1 the baseline values might not be true but instead a “false positive”. My suggestion is to include data of few healthy subjects.

We thank the reviewer for the comment. We understand the reviewer’s concerns regarding the fact that we had only a single individual as control (although we had multiple measurements over multiple visits on this individual and we provide an indication of not only the mean but the full range of values seen during practice). Also, anecdotally we wish to note that the best times for the stacking task of the control participant and the main participant were within 25% of the best time of what can be considered an expert user’s time (the expert user was the researcher who developed the system and had practiced on it extensively during testing) – main best: 446 s, control best: 376 s, expert best ~300 s. Even though this data is anecdotal, we think it gives us confidence that the data we report are not far off the mark.

Ultimately, given this is a case-study, we think this result can only be treated as a proof of concept and is not intended to be generalizable - so issues such as false positives or negatives are not applicable. Moreover, given the current situation with COVID-19, we believe that we will not be able to get this data for several months, and it would not fundamentally alter any of the conclusions in the paper.

We have now explicitly addressed the limitations of our results as needing further study in order to be applied generally. We hope that this additional narrowing of the scope of the generality of our work is sufficient.

Our results add to these prior findings by demonstrating that (i) these interfaces are well-suited for children, and (ii) the improvement in performance over multiple practice sessions is substantial (up to 40-50% reduction in movement times). The child’s performance for the tasks was found to be comparable to that of an adult control participant using a manual joystick. However, given that we only had data from a single child and a single adult, additional studies are needed for assessing the generality of these findings.
